# Neutrophil-to-Lymphocyte Ratio Predicts Early Mortality in Patients with HBV-Related Decompensated Cirrhosis

**DOI:** 10.1155/2016/4394650

**Published:** 2016-02-02

**Authors:** Honggang Zhang, Qinqin Sun, Weilin Mao, Jian Fan, Bo Ye

**Affiliations:** ^1^Department of Anesthesiology, The First Affiliated Hospital, College of Medicine, Zhejiang University, Zhejiang 310003, China; ^2^Department of Urology, The Sixth Affiliated Hospital of Xinjiang Medical University, Xinjiang 830001, China; ^3^Department of Clinical Laboratory, The First Affiliated Hospital, College of Medicine, Zhejiang University, Zhejiang 310003, China

## Abstract

*Background*. The neutrophil-to-lymphocyte ratio (NLR) is an inflammation index that has been shown to independently predict poor clinical outcomes. We aimed to evaluate the clinical value of NLR in the prediction of 30-day mortality in patients with HBV-related decompensated cirrhosis (HBV-DeCi).* Methods*. This was a retrospective cohort study that included 148 patients with HBV-DeCi.* Results*. An elevated NLR was associated with increased severity of liver disease and mortality within 30 days. Multivariate analysis suggested that NLR, similar to the model for end-stage liver disease (MELD) score, is an additional independent predictor of 30-day mortality (*P* < 0.01).* Conclusion*. Our results suggest that a high NLR can be considered a new independent biomarker for predicting 30-day mortality in patients with HBV-DeCi.

## 1. Introduction

All chronic liver diseases can lead to liver cirrhosis (LC). Hepatic decompensation ultimately occurs in cirrhotic patients as a result of progressive portal hypertension, impairment of hepatic biologic functions, or cancer development [1, 2]. Chronic hepatitis B virus (HBV) infection remains a major cause of LC in China, with a yearly incidence of decompensated cirrhosis (DeCi) of 3% [3]. Previous studies have shown that the prognosis of DeCi is usually poor with a 5-year survival rate of only 14–35% under conventional standard care [4, 5]. Although the majority of patients with HBV-related decompensated cirrhosis (HBV-DeCi) can be referred for liver transplantation, the shortage of donor livers and considerable cost make this approach unavailable for most patients at present [6, 7]. Therefore, discovery of a marker associated with HBV-DeCi severity will help improve clinical management to mitigate the high rate of mortality.

Necroinflammation is an essential component of liver pathology in chronic HBV infection. The neutrophil-to-lymphocyte ratio (NLR), a simple and effective marker that reflects the severity of inflammation, is easily calculated from routinely available data. Neutrophilia occurs in chronic inflammation, and lymphopenia is associated with malnutrition and bacterial infection. High NLRs have been shown to predict outcomes in various disease processes including cardiac disease, malignancy, and renal failure [8–10]. More recently, Chen et al. and Liu et al. showed that the NLR measured on hospital admission can serve as an independent predictor of the 3-month mortality rate in patients with acute-on-chronic liver failure (AoCLF) [11, 12]. Furthermore, Biyik et al. found that a greater NLR is associated with an increased risk of long-term death in cirrhotic patients who are at an early advanced stage (mean model for end-stage liver disease (MELD) score 10 and Child-Pugh score 7) [13]. However, currently there are few markers that can predict 30-day mortality after hospital admission of patients with DeCi. In the present study, we hence investigated NLR as a predictor for 1-month mortality in a cohort of HBV-DeCi patients.

## 2. Patients and Methods

This was a retrospective follow-up study of a cohort of 148 consecutive in-patients who were diagnosed with decompensated cirrhosis and met the inclusion and exclusion criteria between January 2014 and January 2015.

For inclusion, patients had to be HBsAg positive, previously diagnosed with HBV-related compensated cirrhosis, and presenting clinical manifestations of decompensated liver disease for the first time. Patients were excluded according to the following criteria: acute hepatitis; hematologic disorder; malignancy such as hepatocellular carcinoma; pregnancy; concurrent infection with HCV, hepatitis D virus, or human immunodeficiency virus; or concurrent autoimmune or other liver diseases.

Decompensation was defined by the appearance of clinical ascites, variceal bleeding, jaundice, or hepatic encephalopathy (HE) [14]. At baseline, for each patient, demographic and clinical data including age, sex, and complications of ascites, variceal bleeding, hepatorenal syndrome (HRS), or HE and clinical course in the hospital were obtained from the medical files and recorded in a specified liver disease pro forma. In our cohort, 120 patients were receiving antiviral therapy, 70 had started antiviral therapy before admission, and 50 had started after admission. Only 28 patients did not receive any antiviral therapy throughout the clinical course for economic reasons. Biochemical parameters including creatinine, liver function tests, white cell and differential counts, and platelet counts were recorded. The NLR was calculated as the absolute neutrophil count divided by the absolute lymphocyte count. A NLR ≥ 5 was considered elevated [15, 16]. In addition, the MELD score and serological indexes (HBsAg, HBeAg, anti-HBc, and HBV DNA levels) were detected at baseline.

The study was performed in accordance with the Declaration of Helsinki, and the procedures were approved by the Ethics Committee of the First Affiliated Hospital of Zhejiang University College of Medicine. The need for written patient consent was waived.

### 2.1. MELD Score

Liver disease severity was evaluated using the MELD score, which includes the patient's serum bilirubin and creatinine levels and the international normalized ratio (INR) for prothrombin time. The MELD score was calculated using an online calculator (http://www.mayoclinic.org/gi-rst/mayomodel7.html).

### 2.2. Statistical Analysis

All continuous variables were expressed as mean ± standard deviation (SD) or median (range), and categorical data were calculated as percentages. The differences in the variables were weighted using analysis of variance (ANOVA) or the Kruskal-Wallis tests. Categorical data were evaluated by the *χ* ^2^-test, as appropriate. Correlations between variables were examined using Spearman's correlation test. Multivariate analysis was performed using Cox proportional hazards regression that weighed all possible clinical factors. The receiver operator characteristic (ROC) curves and the respective areas under the curve (AUCs) were used to assess the ability for the prediction of death. Statistical analyses were performed using the SPSS version 12.0 statistical package (SPSS Inc., Chicago, IL), and a *P* < 0.05 was considered statistically significant.

## 3. Results

### 3.1. Baseline Characteristics of All Patients

**Figure 1:**
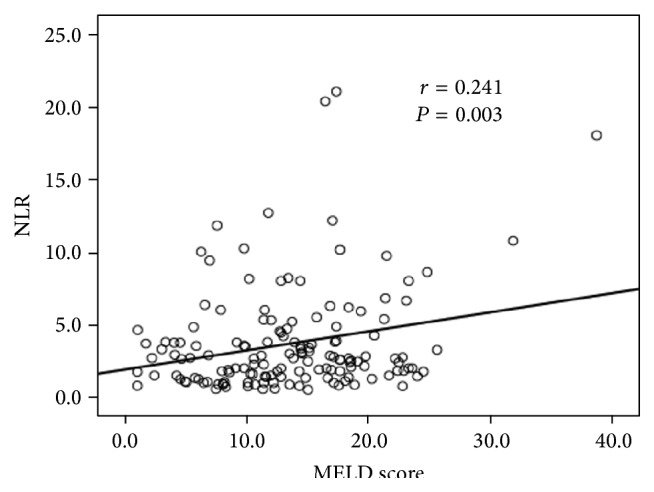
Scatter diagram for correlation analysis, showing a positive correlation between the NLR and MELD score in HBV-DeCi patients.

A total of 148 patients (118 males, mean age: 53.2 ± 11.2 years) with HBV-DeCi were enrolled in the present study. Their baseline characteristics are summarized in Table 1. A significant positive correlation between NLR and MELD score (*r* = 0.241, *P* = 0.003) was detected in patients (Figure 1).

### 3.2. Baseline Characteristics of Patients with Different NLR Levels

HBV-DeCi patients were divided into three groups based on NLR levels: group A (NLR ≤ 2.0), group B (>2.0, but <5.0), and group C (≥5.0). The clinical and laboratory characteristics of the three groups are listed in Table 2. There were significant differences in total protein, serum albumin, serum creatinine, MELD score, and mortality rate among the three groups (*P* = 0.004, *P* = 0.023, *P* = 0.013, *P* = 0.033, and *P* = 0.036, resp.). Moreover, highly elevated NLR levels were associated with higher frequencies of clinical complications such as HRS (*P* = 0.035). No significant differences in ALT, AST, total bilirubin, INR levels, gender, or age were detected. Furthermore, patients in group C had significantly higher white cell and neutrophil counts than patients in groups A and B, whereas the lymphocyte count was lower than those in groups A and B. These data suggest that a higher NLR in DeCi patients could be primarily attributed to increased neutrophil counts and decreased lymphocyte counts.

### 3.3. Relative Risk Factors for 30-Day Mortality

**Figure 2:**
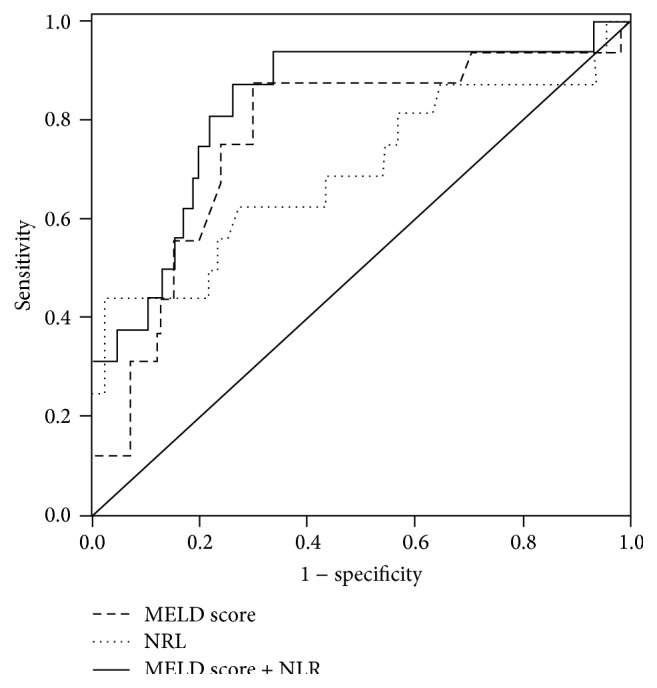
Receiver operating characteristic curve, indicating the relative efficiencies for prediction of 1-month mortality by NLR (…), MELD score (– – –), and their combination (—).

The patients were followed up for a median of 20 days (IQR: 13–80 days). During the follow-up, 16 patients died within 1 month due to upper gastrointestinal bleeding (*n* = 6), HE (*n* = 3), or HRS (*n* = 7). Our findings indicated that an elevated NLR level at admission was followed by an increased 1-month mortality rate, increasing from 4.9% in group A to 10.7% in group B and 22.6% in group C. Univariate and multivariate logistic regression analyses showed that a high NLR was an additional independent risk factor for 1-month mortality in HBV-DeCi patients, similar to a high MELD score (Table 3). To evaluate the ability of NLR and MELD scores to predict mortality, ROC curves were obtained (Figure 2). The AUC values were 0.832 ± 0.062 for the MELD score and 0.736 ± 0.076 for NLR (both *P* < 0.005). When NLR and MELD score were combined, the AUC was 0.891 ± 0.057 (*P* < 0.001). The sensitivity and specificity of the MELD score were 91.2% and 73.4%, respectively, with 29.8% positive values and 98.5% negative predictive values. When the NLR and MELD score were combined, the sensitivity (93.8%) and specificity (81.0%) were significantly improved, with 37.9% positive values and 99.1% negative predictive values.

## 4. Discussion

In the present study, we examined the ability of the blood NLR, a simple marker that reflects the severity of inflammation, to predict 1-month mortality in HBV-DeCi patients. We made two important observations. First, we found that a higher NLR correlated with increased frequency of liver related complications such as HRS. Secondly, we demonstrated that an elevated NLR level can serve as an independent predictor for mortality in HBV-DeCi patients and the risk of mortality increases as the NLR increases.

The MELD score is known to signal risk for 3-month mortality and is used to assign priorities in the transplant waiting list for cadaveric livers [17]. Our previous study reported that the MELD score was associated with the prognosis of patients with AoCLF [18]. In the present retrospective study of HBV-DeCi, a significant positive association was found between the NLR and the MELD score. The NLR can be used to predict HBV-DeCi patients' mortality, although the predictive power of NLR was relatively lower than that of the MELD score. However, the NLR involves only two markers, which makes it simpler and easier to calculate than the MELD score. A combination of NLR with the MELD score augmented the predictive power.

The physiopathologic association between an elevated NLR and poor prognosis is complex and remains to be elucidated. Chronic HBV infection can cause ongoing liver injury. Repeated repair of the damaged liver parenchyma could lead to fibrosis and cirrhosis. A cirrhosis patient who has advanced to the decompensatory stage can be very vulnerable to new insults including reactivation of HBV replication in the infected liver. Elevated neutrophil counts are a key component of an increased NLR. In our study, we noted that there were marked increases in the white cell and neutrophil counts with a higher NLR compared with those with lower NLR levels. We reasoned that newly intensified hepatic inflammation in DeCi patients during flare-ups of liver injury triggered a large neutrophil response. In addition, lymphocyte counts were lower with a higher NLR compared with those with lower NRLs in our study. One possible explanation for the reduced number of lymphocytes in peripheral blood is that lymphocytes were largely recruited to the liver to participate in the necroinflammation. A previous investigation showed that the intrahepatic CD8+ T-cell number was approximately 50-fold greater in AoCLF patients compared to normal individuals [19]. Lymphopenia was once an indication of malnutrition and poor response of immunity in patients with chronic liver disease [20, 21]. Our results were in accordance with those of Berres et al., who found that a lower lymphocyte count was associated with mortality in patients with end-stage cirrhosis listed for liver transplantation [22]. Our findings indicate that the higher NLR mainly resulted from increased neutrophil counts and decreased lymphocyte counts. Therefore, a high neutrophil count together with a low lymphocyte count may reflect the severity and progress of liver injury in HBV-DeCi patients.

## 5. Conclusion

This study of the NLR in hospitalized patients with HBV-DeCi showed that an elevated NLR was associated with the severity of HBV-DeCi, and the NLR can function as an additional marker for predicting 1-month mortality in this cohort. However, possible application of our findings is limited by the retrospective nature of the study and the relatively small number of patients included. Clearly, further prospective studies are warranted to confirm our findings.

## Figures and Tables

**Table 1:** Baseline demographic and clinical characteristics of cohort.

	HBV-DeCi patients (*n* = 148)
Gender (male/female)	118/30
Age (y)	53.2 ± 11.2
Total protein (g/L)	61.0 ± 8.2
Albumin (g/L)	30.4 ± 5.9
ALT (U/L)	30.5 (17.0–55.0)
AST (U/L)	48.0 (29.0–79.0)
Total bilirubin (*μ*mol/L)	50.5 (23.5–117.0)
INR	1.50 ± 0.40
Creatinine (mmol/L)	72.5 (60.0–88.0)
Platelet count (×10^9^/L)	65.5 (38.0–113.5)
White blood cell count (×10^9^/L)	4.35 (2.90–5.90)
Neutrophil count (×10^9^/L)	2.40 (1.50–3.70)
Lymphocyte count (×10^9^/L)	0.90 (0.60–1.40)
NLR	2.67 (1.46–4.39)
MELD score	13.4 ± 6.7
HBsAg positive	148
HBeAg positive	80
HBcAb IgM positive	0
HBV DNA positive	148
HE (*n*)	3
Ascites (*n*)	107
Variceal bleeding (*n*)	40
HRS (*n*)	29

Data are expressed as *n*, mean ± SD, or median (interquartile range).

ALT, alanine aminotransferase; AST, aspartate aminotransferase; INR, international normalized ratio; NLR, neutrophil-to-lymphocyte ratio; MELD score, model for end-stage liver disease score; HE, hepatic encephalopathy; HRS, hepatorenal syndrome.

**Table 2:** Clinical and laboratory characteristics in patients with different neutrophil-to-lymphocyte ratio (NLR) at admission.

	Group A	Group B	Group C	*P*
	(NLR ≤ 2.0, *n* = 61)	(2.0 < NLR < 5.0, *n* = 56)	(NLR ≥ 5.0, *n* = 31)	
Age (y)	53.0 ± 10.1	53.5 ± 12.7	52.8 ± 10.7	0.946
Gender (male/female)	47/14	47/9	24/7	0.611
Total protein (g/L)	61.7 ± 6.4	62.6 ± 8.0	56.8 ± 10.4	0.004
Albumin (g/L)	29.6 ± 5.9	32.0 ± 5.9	28.9 ± 5.4	0.023
ALT (U/L)	44.5 ± 44.1	59.4 ± 93.2	65.2 ± 72.5	0.379
AST (U/L)	57.5 ± 39.0	81.0 ± 118.4	94.6 ± 98.5	0.182
INR	1.46 ± 0.29	1.50 ± 0.48	1.56 ± 0.41	0.471
Creatinine (mmol/L)	74.9 ± 19.9	77.4 ± 43.4	100.4 ± 61.1	0.013
Total bilirubin (*μ*mol/L)	79.8 ± 91.5	98.3 ± 107.8	123.3 ± 162.9.37	0.233
MELD score	12.46 ± 1.73	13.88 ± 6.15	16.14 ± 7.84	0.033
White blood cell count (×10^9^/L)	3.51 ± 6.22	4.62 ± 2.09	10.00 ± 8.66	<0.001
Neutrophil count (×10^9^/L)	1.63 ± 0.79	3.00 ± 1.38	8.22 ± 7.36	<0.001
Lymphocyte count (×10^9^/L)	1.33 ± 0.71	0.95 ± 0.45	0.88 ± 0.81	0.001
Ascites (yes/no)	40/21	45/11	22/9	0.200
HRS (yes/no)	8/53	10/46	11/20	0.035
HE (yes/no)	0/61	1/55	2/29	—
Mortality (yes/no)	3/58	6/50	7/24	0.036

Data are expressed as *n* or mean ± SD.

ALT, alanine aminotransferase; AST, aspartate aminotransferase; INR, international normalized ratio; NLR, neutrophil-to-lymphocyte ratio; MELD score, model for end-stage liver disease score; HE, hepatic encephalopathy; HRS, hepatorenal syndrome.

**Table 3:** Cox proportional hazards analysis for predictors of mortality.

	Univariate			Multivariate		
	Odds ratio	95% CI	*P*	Odds ratio	95% CI	*P*
NLR	1.226	1.071–1.402	0.003	1.296	1.047–1.604	0.017
MELD score	1.245	1.115–1.391	<0.001	1.274	1.031–1.573	0.025
Age (y)	1.003	1.000–1.051	0.896	—	—	0.917
Albumin (g/L)	0.995	0.908–1.090	0.442	—	—	0.180
Total protein (g/L)	0.996	0.930–1.067	0.910	—	—	0.604
White blood cell count (×10^9^/L)	1.031	0.900–1.118	0.663	—	—	0.223

CI, confidence interval; NLR, neutrophil-to-lymphocyte ratio; MELD score, model for end-stage liver disease score.

## Abbreviations

HBV: Hepatitis B virus
CI: Confidence interval
DeCi: Decompensated cirrhosis
NLR: Neutrophil-to-lymphocyte ratio
ALT: Alanine aminotransferase
AST: Aspartate aminotransferase
MELD score: Model for end-stage liver disease score
INR: International normalized ratio
HE: Hepatic encephalopathy
HRS: Hepatorenal syndrome
ROC: Receiver operating characteristic
AUCs: Areas under the curve.
